# Disruption of the Contents of Endogenous Hormones Cause Pollen Development Obstruction and Abortion in Male-Sterile Hybrid Lily Populations

**DOI:** 10.3390/plants12223804

**Published:** 2023-11-08

**Authors:** Wenjie Jia, Xiang Li, Rui Wang, Qing Duan, Junna He, Junping Gao, Jihua Wang

**Affiliations:** 1 Beijing Key Laboratory of Development and Quality Control of Ornamental Crops, College of Horticulture, China Agricultural University, Beijing 100193, China; jiawenjie917917@163.com (W.J.); b20213170896@cau.edu.cn (R.W.);; 2 Flower Research Institute, Yunnan Academy of Agriculture Sciences, Kunming 650000, China; leexiang93@163.com (X.L.); duanqing123@126.com (Q.D.)

**Keywords:** hybrid lily population, male−sterile, pollen abortion, hormone

## Abstract

Lilies are well−known flowers with large anthers and a high quantity of pollen that easily contaminates clothing and tepals. The anthers need to be artificially removed, leading to production problems. Cultivating male−sterile or pollen−free lilies could solve these problems. The key period of male sterility in a specific male−sterile hybrid lily population was determined through cytological observation. The contents of hormones, soluble sugar, soluble protein, and proline were determined by high−performance liquid chromatography, tandem mass spectrometry and colorimetry. Transcriptome sequencing was used to identify the genes with altered expression. The key period of male sterility was determined to be the microspore mother and tetrad stages. The hormone contents were abnormal in the sterile line compared with the fertile line. The indole−3−acetic acid (IAA) content was higher in the sterile line than in the fertile line at all stages, while the gibberellic acid 4 (GA4) content showed the opposite result. Abscisic acid (ABA) accumulated in the sterile line in both the microspore mother and tetrad stages, and the zeatin riboside (ZR) content in the sterile line increased at the microspore mother stage but decreased at the tetrad stage. The contents of soluble sugar, soluble protein and proline were higher in the fertile line than in the sterile line. Genes involved in auxin and ABA synthesis and signalling pathways were highly expressed in the male−sterile line. Our data suggested that abnormal contents of hormones in the microspore mother and tetrad stages resulted in pollen abortion in a male−sterile hybrid lily population, which indicated that the hormone balance in specific stages plays critical functions in pollen development in lilies.

## 1. Introduction

Lilies are perennial bulb flowers in the family Liliaceae. Because of their beautiful flower shape, gorgeous colour and pleasant aroma, lilies can be used as cut flowers, potted flowers, and garden flowers. Lilies are deeply loved by people and are presently one of the most popular types of cut flowers on the international market. As a major commodity flower around the world, lilies are widely planted in China and occupy an enormous market share [1,2,3,4]. Because lily pollen easily contaminates clothing and cleaning the clothing after contamination is inconvenient, lilies sold in the market are generally artificially emasculated via a complex process that wastes considerable manpower and resources and brings considerable trouble to their production [5,6,7]. Therefore, cultivating lilies that are male−sterile and pollen−free is a key direction for breeding in the future.

Male sterility (MS) is a common biological phenomenon in abnormal flower organ development in individual plants that results in an inability to produce normal pollen or impaired pollen function and the inability to carry out effective pollination and fertilization [8]. Recently, many studies have shown that hormones and endogenous substances can cause male sterility in plants, and these studies have been applied to the model plants *Arabidopsis thaliana* [9,10,11,12], rice [13], wheat [14], and *Brassica napus* [15]. Decreasing auxin levels is essential to promote anther dehiscence and pollen maturation during pollen mitosis, especially during late stamen development [16]. Exogenous application of abscisic acid (ABA) prior to heat stress increased sucrose transport and accelerated sucrose metabolism to maintain the carbon balance and energy homeostasis, which reduced pollen sterility caused by heat stress in Zhefu802 material [17]. Mutations affecting gibberellin (GA) biosynthesis in *Arabidopsis thaliana*, such as *ga1−3* mutations, exhibit male−sterile phenotypes, such as failure of stamen filament elongation and anther dehiscence [18]. High levels of cytokinin disrupt the anther respiratory system and energy metabolism, resulting in impaired microspore development and abortion [19].

When plant energy metabolism is abnormal, the process of microspore formation is aborted, forming inactive pollen or no pollen at all [20,21]. As an important source of energy, sugar plays a significant role in the male reproductive process of plants. Defects in sugar metabolism during anther development led to male sterility [22]. In the presence of sugars derived from starch, mature pollen can germinate smoothly, facilitating the formation of pollen tube cell walls [23]. In higher plants, proline is synthesized from the reduction in glutamate through the consumption of ATP and NADPH [24]. The synthesis of proline in developing microspores and mature pollen grains is essential for ensuring pollen fertility, and disruption of proline synthesis results in sterility in Arabidopsis gametophytic development [25]. Previous research has indicated that the accumulation of MDA was significantly higher in the sterile line than that of fertile lines at the tetrad stage during the development of lily anthers [26].

There are numerous factors contributing to male sterility in plants, yet studies on male sterility in ornamental horticultural plants such as the lily remain scarce. Male sterility can arise from changes in the content and balance of endogenous hormones during stamen development, abnormalities in the inner wall structure of pollen grains, and premature degradation of the tapetum layer [25,27,28]. Disturbances in material metabolism and energy deficits eventually lead to male sterility [20,29]. Genes associated with the hormonal regulation of pollen development were analyzed using transcriptome sequencing, which will provide knowledge for us understanding the mechanisms of lily pollen sterility.

In this research, to investigate the effects of hormone content and endogenous substances during this critical period of pollen development, parental individuals including maternal (F) and paternal (M), hybrid fertile offspring (HF), and hybrid sterile offspring (HS) of a male sterile hybrid population of Oriental Lily were utilized, which were bred for many years by the Institute of Floriculture, Yunnan Academy of Agricultural Sciences. The impacts of hormones and endogenous substances on male sterility in lilies were studied by comparing their contents between the parents (M and F), hybrid sterile offspring (HS), and hybrid fertile offspring (HF) in different anther development stages. This study further revealed the hormone gene regulatory network associated with anther and pollen development by examining their expression in the offspring of the male−sterile hybrid population using RNA−seq. These results could establish a foundation for the development of exogenous hormone regulation for lily pollen fertility and could also contribute to revealing the biological mechanisms governing plant reproduction.

## 2. Results

### 2.1. Observation of the Phenotype and Histological Microstructure of Lily Anthers

**Figure 1:**
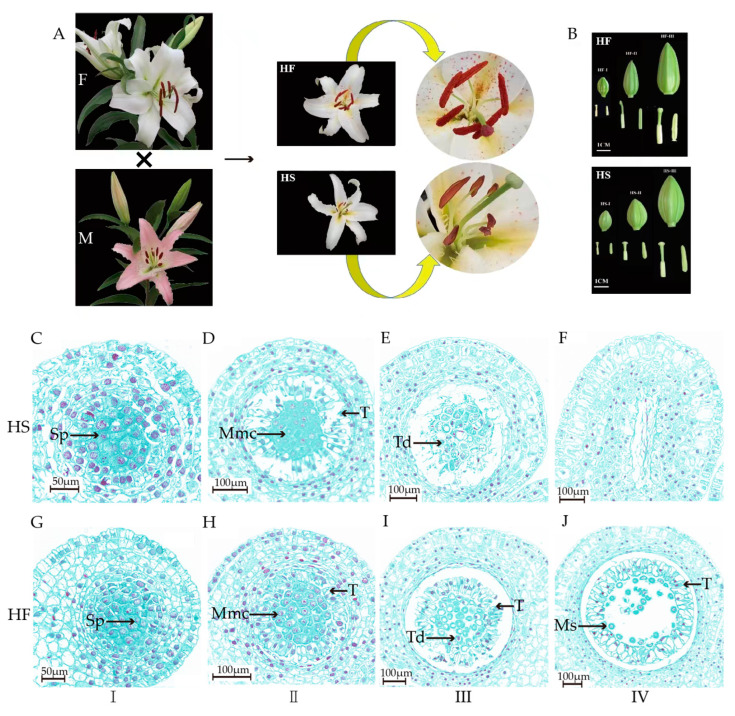
Phenotype and cytological observation of the HS and HF lines during different anther growth stages. (**A**): The morphology and phenotype of sterile and fertile lilies. (**B**): Flower bud of the HS and HF lines in different anther growth stages. (**C**–**F**): Cytological observation of the HS lines during different anther growth stages. (**G**–**J**): Cytological observation of the HF lines during different anther growth stages. F, maternal; M, paternal; HS, sterile hybrid; HF, fertile hybrid; I, sporogenous cell stage; II, microspore mother cell stage; III, tetrad stage; IV, microspore stage; Sp, sporogenous cell; Mmc, microspore mother cell; Td, tetrad of a microspore; T, tapetum layer; Ms, microspore.

The phenotype of hybrid fertile offspring (HF), and hybrid sterile offspring (HS) of a male sterile hybrid population of Oriental Lily was observed and is shown in Figure 1A,B. The buds, tepals, and anthers both grow normally in HF and HS lines; however, there is no pollen released from the anthers in HS. Then, anther histology was observed in cell sections, which showed that the anther cross section of HS was different from that of HF (Figure 1C–J). In the sporogenous stage (I), the microspores of both strains developed normally, and the anther cross sections of HS and HF were not significantly different at this stage. In the microspore mother cell stage (II), the stage of HS tapetal cells arrived early, tapetal cell development stopped, and the tapetal began to degenerate gradually, while HF microspore mother cells had complete nuclei and thick cytoplasm that was tightly surrounded by tapetal cells. In the tetrad stage (III), the HS microspore mother cells showed a high degree of vacuolization, and the contents from degradation of the tapetum cells filled the anther chamber and wrapped the microspore mother cells, inhibiting them from entering the tetrad stage normally. At the same stage, HF single microspore was formed by meiosis of microspore mother cells produced from tetrad formation connected by callose walls. At the same time, the middle layer cells disappeared, and the tapetum cells gradually enlarged. In the microspore stage (IV), the microspores became highly vacuolated and gradually disappeared, the cell wall became thinner in the HS lines, while the pollen sac was full of mature pollen grains in the HF lines.

### 2.2. The Endogenous Hormone Content Changed during Anther Development in Lily

**Figure 2:**
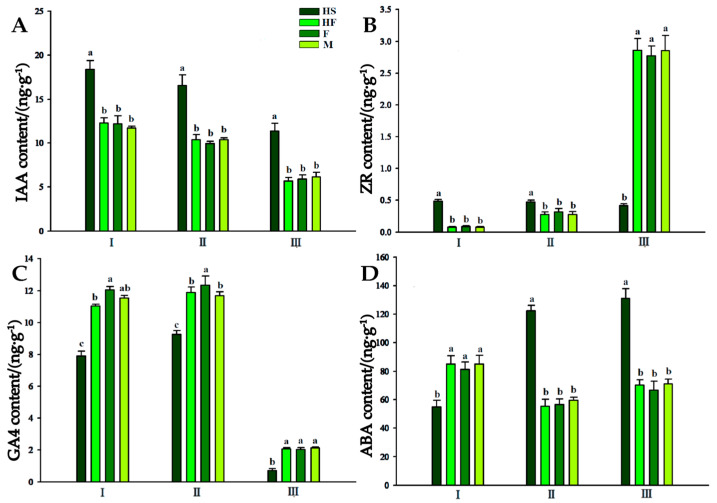
Changes in hormone contents in the anthers of the male−sterile line and fertile lines of Lilium at different developmental stages. (**A**): Indole−3−acetic acid (IAA) content in the anthers of the male−sterile line and fertile lines of Lilium. (**B**): Zeatin riboside (ZR) content in the anthers of the male−sterile line and fertile lines of Lilium. (**C**): Gibberellic acid 4 (GA4) content in the anthers of the male−sterile line and fertile lines of Lilium. (**D**): Abscisic acid (ABA) content in the anthers of the male−sterile line and fertile lines of Lilium. I, sporogenous cell stage; II, microspore mother cell stage; III, tetrad stage. HS, sterile hybrid; HF, fertile hybrid; F, maternal; M, paternal. Two independent experiments were performed, each with three technical replicates, and one experimental result is shown. Different letters indicate significant differences among treatments at *p* < 0.05 (one−way ANOV A followed by Dunnett’s test). The error bars indicate the mean ± SD (*n* = 3).

During anther development, the indole−3−acetic acid (IAA) content in HS, HF, F (maternal), and M (paternal) anthers showed a gradually decreasing trend. The IAA content in HS was significantly higher than that in the fertile lines HF, M, and F in the sporogenous cell stage, microspore mother cell stage, and tetrad stage, respectively, with an IAA content in HS that was 1.52 times, 1.62 times, and 1.92 times of that in these three stages, respectively (Figure 2A).

The zeatin riboside (ZR) content in anthers of sterile HS and fertile HF, M, and F was significantly different in the three stages, and ZR content in HS was significantly higher than that in HF, M, and F in the sporogenous cell stage. The ZR content in the fertile lines in the microspore mother cell stage was higher, but it was still lower than that in the HS line. In the tetrad stage, the ZR content in the fertile lines (HF, M, and F) continued to rise and was significantly higher than that in HS (Figure 2B). 

The gibberellic acid 4 (GA4) content in the lily anthers showed a similar trend in the sterile line (HS) and fertile lines (Figure 2C), rising first and then decreasing. The microspore mother cell stage was the turning point and the peak period of GA4, and the GA4 content in the anthers of the fertile lines was significantly higher than that in the sterile line in all three stages.

The content of abscisic acid (ABA) in the anthers of the fertile lines (HF, M, and F) decreased first and then increased. However, the ABA content in the sterile line (HS) increased gradually during the three periods. Amongst them, the ABA content in the sterile line (HS) in the sporogenous cell stage was significantly lower than that in the fertile lines, while the ABA content in the sterile line (HS) in the latter two stages was significantly higher than that in the fertile lines (Figure 2D).

### 2.3. Endogenous Substance Content Determination

**Figure 3:**
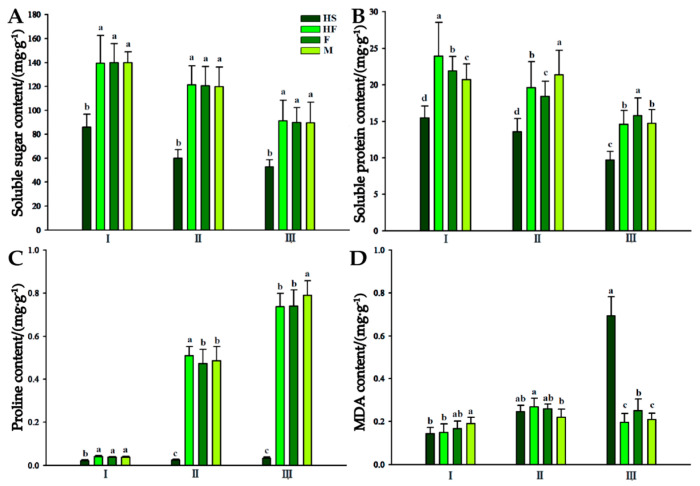
Changes in endogenous substance contents in the anthers of the male−sterile line and fertile lines of Lilium. (**A**): Soluble sugar content in the anthers of the male−sterile line and fertile lines of Lilium. (**B**): Soluble protein content in the anthers of the male−sterile line and fertile lines of Lilium. (**C**): Proline content in the anthers of the male−sterile line and fertile lines of Lilium. (**D**): MDA content in the anthers of the male−sterile line and fertile lines of Lilium. I, sporogenous cell stage; II, microspore mother cell stage; III, tetrad stage; HS, sterile hybrid; HF, fertile hybrid; F, maternal; M, paternal. Two independent experiments were performed, each with three technical replicates, and one experimental result is shown. Different letters indicate significant differences among treatments at *p* < 0.05 (one−way ANOV A followed by Dunnett’s test). The error bars indicate the mean ± SD (*n* = 3).

During anther development, the content of soluble sugar in the sterile line (HS) and fertile lines (HF, M, and F) showed a gradually decreasing trend, and the content of soluble sugar in the anther of the sterile line (HS) was significantly lower than that in the fertile lines at the sporogenous cell stage, microspore mother cell stage, and tetrad stage (Figure 3A). There was no significant difference in the content of soluble sugar amongst the HF, M, and F fertile lines at each developmental stage.

The trend of variation in soluble protein content in the anthers of Lilium in the two lines was similar to that of soluble sugar content, which showed a gradually decreasing trend. There were significant differences in soluble protein content between the sterile line (HS) and fertile lines (HF, M, and F) in the three developmental stages. The soluble protein content in the fertile lines in all developmental stages was significantly higher than that in the male−sterile line at the same period, and the mean soluble protein content in the three fertile lines increased by 43.63%, 45.94% and 55.58%, respectively, compared with that in the male−sterile line at each stage (Figure 3B).

The proline content in lily anthers essentially increased as they progressed through their growth stages (Figure 3C). The proline content in the sterile line (HS) did not increase significantly and remained at a low level during the three developmental stages but increased continuously in the fertile lines (HF, M, and F). The proline content of the fertile lines in the tetrad stage was 0.74 mg·g^−1^, 0.74 mg·g^−1^, and 0.79 mg·g^−1^. The mean value was 1.54 times of that at the microspore mother stage (0.49 mg·g^−1^) and that at the sporogenous cell stage (0.04 mg·g^−1^) (Figure 3C).

In the three stages of anther development, the MDA content in the fertile lines presented an increasing trend after decreasing first, and the peak MDA content in the microspore mother cells of HF, M, and F was 0.27 mg·g^−1^, 0.26 mg·g^−1^, and 0.22 mg·g^−1^, respectively. The MDA content in the sterile line showed a slightly increasing trend from the sporogenous cell stage to the microspore mother cell stage but significantly increased in the tetrad stage, reaching 0.69 mg·g^−1^, which was 3.54 times higher than that in the microspore mother cell stage and significantly higher than that in fertile lines in the same time period (Figure 3D).

### 2.4. Relationship between Endogenous Substances and Endogenous Hormones during Anther Development in Lilium

**Figure 4:**
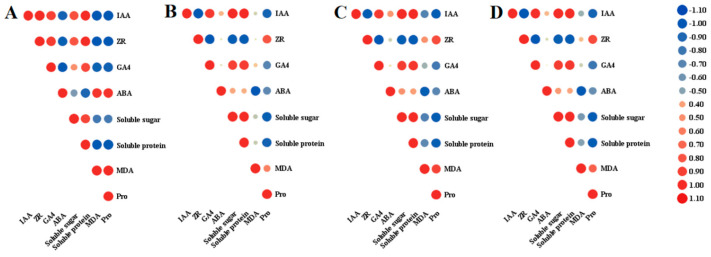
Correlation between endogenous hormones and endogenous substances during anther development in lily. (**A**): The correlation between endogenous hormones and substances during anther development in hybrid sterile offspring (HS). (**B**): The correlation between endogenous hormones and substances during anther development in hybrid fertile offspring (HF). (**C**): The correlation between endogenous hormones and substances during anther development in the hybrid mother (F). (**D**): The correlation between endogenous hormones and substances during anther development in the hybrid male (M).

It can be seen from Figure 4 that different endogenous hormones have different correlations with the contents of soluble sugar, soluble protein, proline, and MDA due to their different functions. In the HS line (Figure 4A), the soluble sugar and protein contents and IAA, ZR, and GA4 contents were positively correlated with each other and negatively correlated with the MDA and proline contents. Conversely, the ABA content was negatively correlated with the sugar and proline contents and positively correlated with the MDA and proline contents. In the three fertile lines (HF, M, and F) shown in Figure 4B–D, the correlation between endogenous hormone and endogenous substance content was basically the same. IAA, GA4, and ABA were positively correlated with soluble sugar and protein content, ABA was not significantly correlated with sugar or protein content, and ZR showed a negative correlation. In contrast, IAA, GA4, and ABA were negatively correlated with MDA and proline content, while ZR was positively correlated with MDA and proline content. MDA had a special correlation with the contents of various hormones, amongst which MDA had no significant correlation with the contents of IAA, ZR, and GA4 but had a significant negative correlation with ABA.

### 2.5. Screening of Related Genes

**Figure 5:**
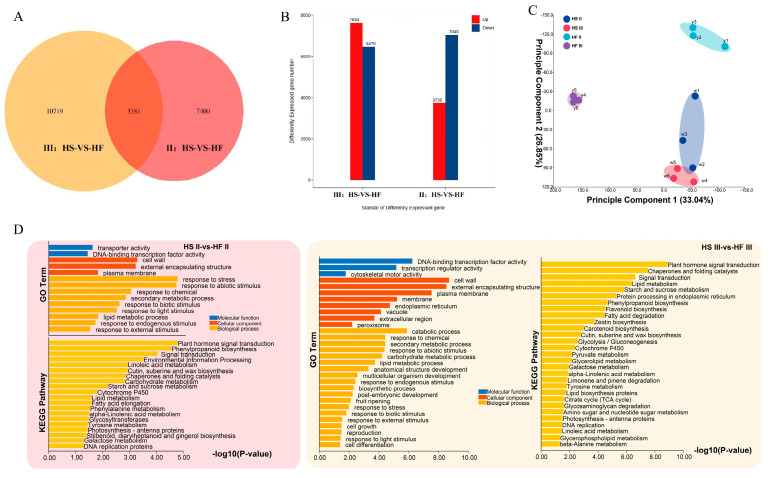
Analysis of differences in the transcriptomes of sterile and fertile lines of lilies. (**A**) Number of differentially expressed genes (DEGs) and common DEGs between HS and HF in the II stage and III stage. (**B**) The number of and changes in DEGs between HS and HF in the II stage and III stage. (**C**) Principal component analysis (PCA) was used to examine the correlation between each sample. (**D**) GO and KEGG enrichment analysis were performed on the differentially expressed genes (DEGs) at two developmental stages. II, microspore mother cell stage; III, tetrad stage; HS, sterile hybrid; HF, fertile hybrid.

Through cytological analyses of the sterile line and fertile lines and determination of hormones and endogenous substances, it was revealed that the microspore mother cell stage and tetrad stage were the key stages of male sterility in lilies. Transcriptome sequencing was performed on the sterile line (HS) and fertile line (HF) in the microspore mother stage and tetrad stage, respectively, with three replicates each, and 12 samples were collected (II−HS1, II−HS2, and II−HS3; II−HF1, II−HF2, and II−HF3; III−HS1, III−HS2, and III−HS3; III−HF1, III−HF2, and III−HF3), yielding 84.14 G of clean data. The effective data volume of each sample ranged from 6.72 to 7.17 G, the Q30 base distribution ranged from 92.41 to 93.22%, and the average GC content was 49.33%. The total length of the 45,244 unigene samples was 46,060,343 bp, and the average length was 1018.04 bp. The results of the Unigene database annotation were as follows: 21,087 (46.61%) genes were annotated to the NR library, 16,143 (35.68%) to the SwissProt library, 4508 (9.96%) to the KEGG library, 12,266 (27.11%) to the KOG library, 19,444 (42.98%) genes were annotated to the eggNOG library, 13,924 (30.78%) genes were annotated to the GO library, and 14,256 (31.51%) genes were annotated to the Pfam library. reads were compared to unigenes with a ratio ranging from 90.03 to 93.58%. There were two different groups. Group 1 was the sterile line (HS) compared with the fertile line (HF) at the microspore mother stage and Group 2 was the sterile line (HS) compared with the fertile line (HF) at the tetrad stage. Amongst them, 3381 differentially expressed genes (DEGs) were shared in these two comparisons between the microspore mother stage and tetrad stage, while the numbers of differentially expressed genes detected were 7400 and 10,719, respectively (Figure 5A). There were 3736 upregulated genes and 7045 downregulated genes in Group 1 and 7630 upregulated genes and 6470 downregulated genes in Group 2 (Figure 5B). 

Through PCA analysis (Figure 5C), we found a strong correlation among the three biological replicates within the same group. Additionally, the distance between the two different time periods of the HS group was close, suggesting minimal differences within the HS group between these two stages. In contrast, the distances between the two stages of the HF group and the HS group were greater, indicating larger differences within the HF groups and a greater separation between the two stages. Gene Ontology (GO) enrichment analysis and Kyoto Encyclopedia of Genes and Genomes (KEGG) analysis were used to classify the potential function of the DEGs involved in these two stages. The results of GO enrichment analysis showed that highly enriched pathways were the DNA−binding transcription factor activity, response to stress or abiotic stimulus, and carbohydrate metabolism process (Figure 5D). Based on the KEGG database, the highly enriched pathways were plant hormone signal transduction, carbohydrate metabolism process, and starch and sucrose metabolism (Figure 5D). These results were related to sugar metabolism, proline biosynthesis and degradation, and the hormone pathway, which were consistent with the hormone, protein and proline concentration examination shown in Figure 2 and Figure 3.

**Figure 6:**
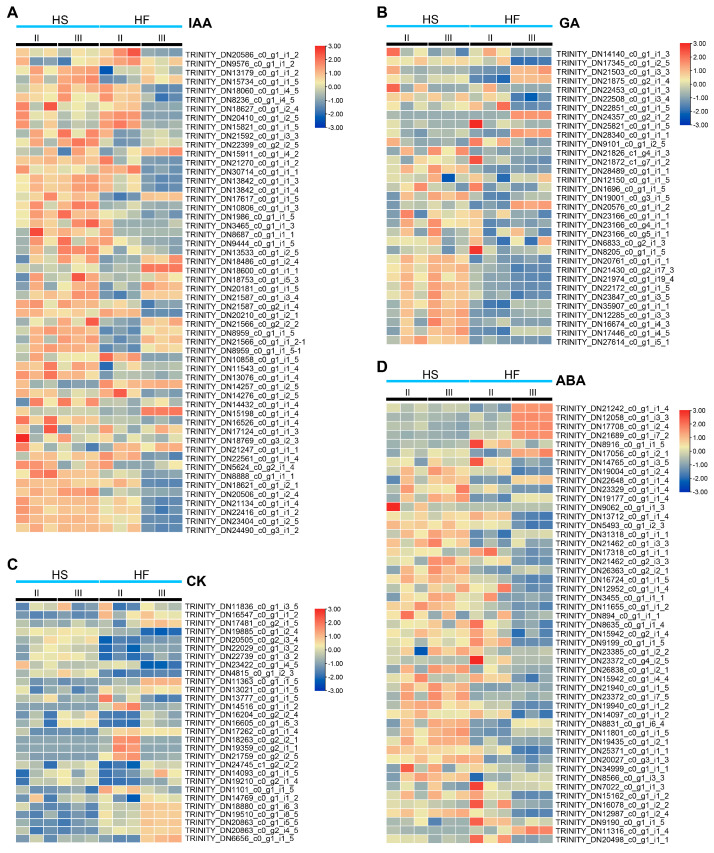
Heat maps of hormone−related genes in the microspore mother stage (II) and tetrad stage (III) of the lily sterile line (HS) and fertile line (HF). (**A**) Heat maps of DEGs related to the auxin pathway. (**B**) Heat maps of DEGs related to the gibberellin acid (GA) pathway. (**C**) Heat maps of DEGs related to the cytokinin (CK) pathway; (**D**) Heat maps of DEGs related to the abscisic acid (ABA) pathway.

The plant hormones auxin, cytokinin, gibberellin, and abscisic acid are the key regulatory factors of plant reproduction and development. By annotating the transcript with the KEGG pathway, we analysed the genes in the above plant hormone metabolism and signal transmission pathways. According to the heatmap of hormone−related genes screened by transcriptomic sequencing, the fertile line (HF) and infertile line (HS) had good consistency and high confidence. There were 59 unigenes associated with IAA synthesis and signal transduction (Figure 6A), 29 unigenes associated with cytokinin (CK) synthesis and signal transduction (Figure 6B), and 33 unigenes associated with GA synthesis and signal transduction (Figure 6C) at the microspore mother stage and tetrad stage. Forty−nine unigenes were associated with ABA synthesis and signal transduction (Figure 6D). The expression levels of auxin and ABA pathway genes in the fertile line (HF) and sterile line (HS) were significantly different in the microspore mother stage (II) and tetrad stage (III). The expression results of auxin and ABA were consistent with those contents of auxin and ABA shown in Figure 2.

### 2.6. Auxin Synthesis−Related Genes Regulate Male Sterility

**Figure 7:**
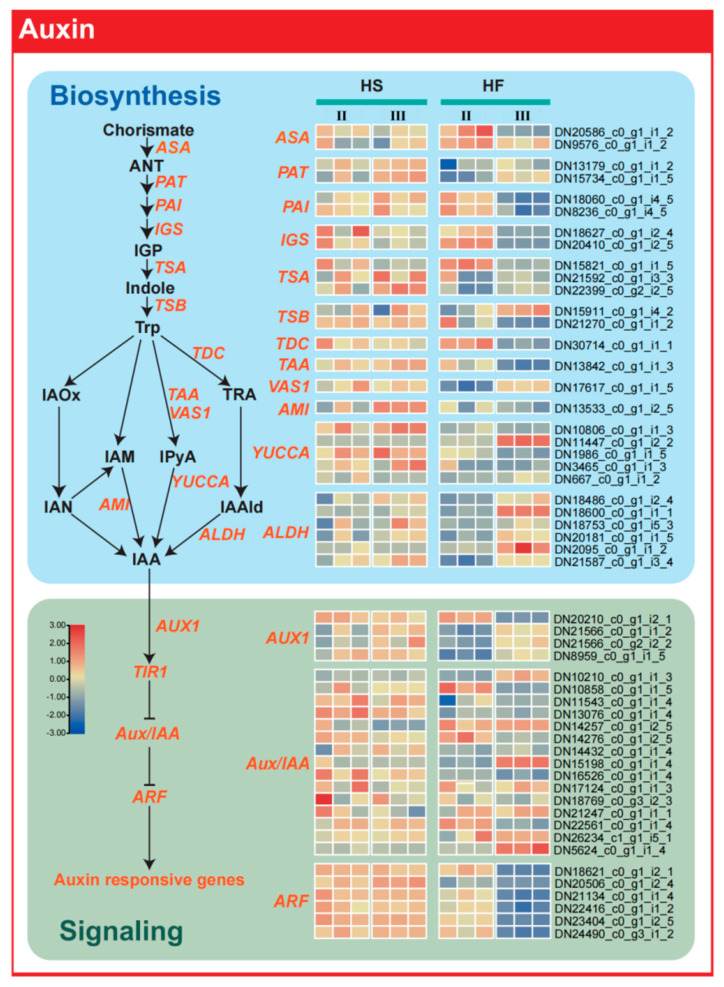
Differences in the expression of genes involved in auxin synthesis and signalling pathways in the fertile line and sterile line during anther development stages. II, microspore mother cell stage; III, tetrad stage; HS, sterile hybrid; HF, fertile hybrid.

In the auxin synthesis pathway, we found that the expression of *TAA* and *indole−3−pyruvate monooxygenase* (*YUCCA*), two key enzymes involved in the conversion of tryptophan into IAA, was always high in the male−sterile line (HS), while in the fertile line (HF), the expression of these two genes was initially high and then decreased (Figure 7). This may be the reason for the difference in IAA content in lily anthers detected with or without pollen. Analysis of auxin signal transduction gene expression also showed that ARF transcription factor expression was significantly different amongst fertile line (HF) plants, and ARF gene expression was significantly decreased in fertile line (HF) plants in the tetrad stage compared with the microspore mother stage but not in the sterile line (HS).

### 2.7. Abscisic Acid Synthesis−Related Genes Regulate Male Sterility

**Figure 8:**
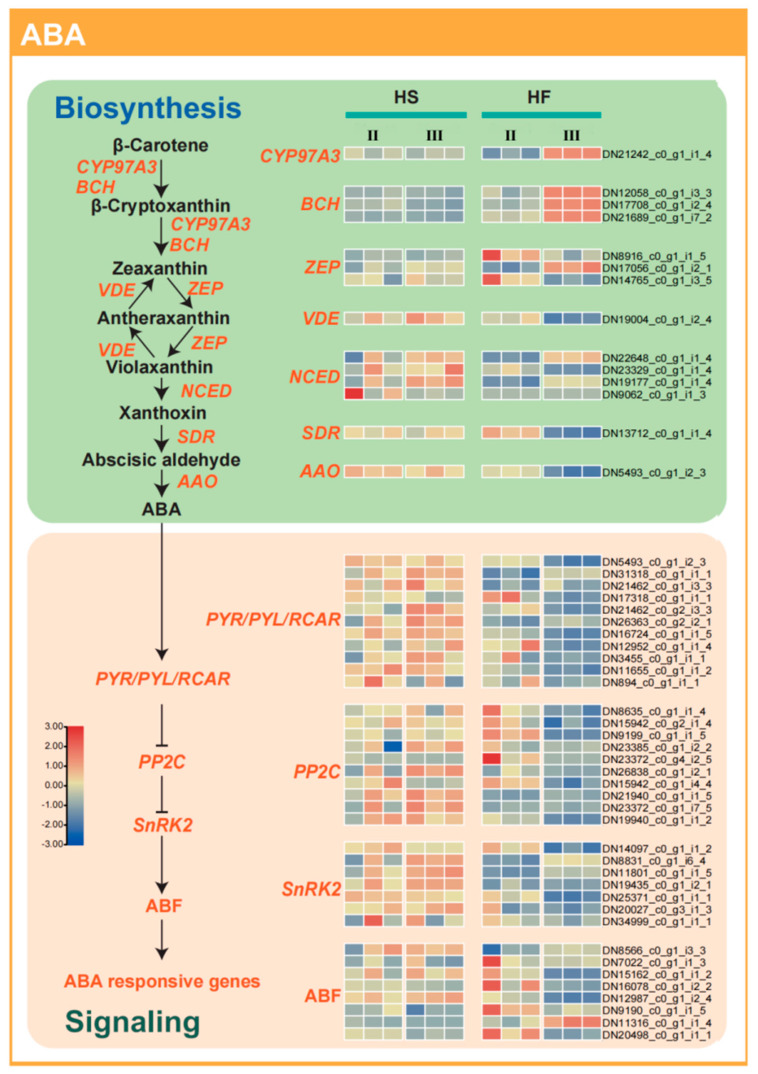
Differences in the expression of genes involved in ABA synthesis and signalling pathways in the fertile line and sterile line during anther development stages. II, microspore mother cell stage; III, tetrad stage; HS, sterile hybrid; HF, fertile hybrid.

An expression pattern similar to that of the auxin pathway was found for the abscisic acid pathway (Figure 8). In the sterile line (HS) plants, the expression of key genes related to abscisic acid synthesis such as *NCED*, *SDR*, and *AAO* remained high in the microspore mother stage and tetrad stage, while the expression of these genes in the fertile line (HF) plants decreased significantly in the tetrad stage. Amongst the genes related to abscisic acid signal transduction, it was also reflected that in the tetrad stage, the expression of the abscisic acid receptor gene *PYR/PYL/RCARs* in fertile line (HF) plants was significantly decreased compared with that in the microspore mother stage, and signal transduction genes such as *PP2Cs* and *SnRK2s* also showed a similar trend. In the sterile line (HS) plants, the expression of the abscisic acid receptor gene was significantly decreased. The expression of these genes increased in the tetrad stage.

### 2.8. Regulation of the Expression of Genes Associated with Male Sterility

By comparing the differences in unigene expression between the microspore mother stage and tetrad stage, we further selected unigenes with differential expression of 4 hormones at these two developmental stages as candidate unigenes. They mainly involve auxin response factor, indole−3−pyruvate monooxygenase (YUCCA), cytokinin synthase and pseudohistidine−containing phosphotransfer protein, gibberellin−regulated protein, gibberellin 2−beta−dioxygenase, gibberellin receptor, abscisic acid receptor, abscisic−aldehyde oxidase, etc. KEGG annotations of gene function are shown in Table 1.

**Figure 9:**
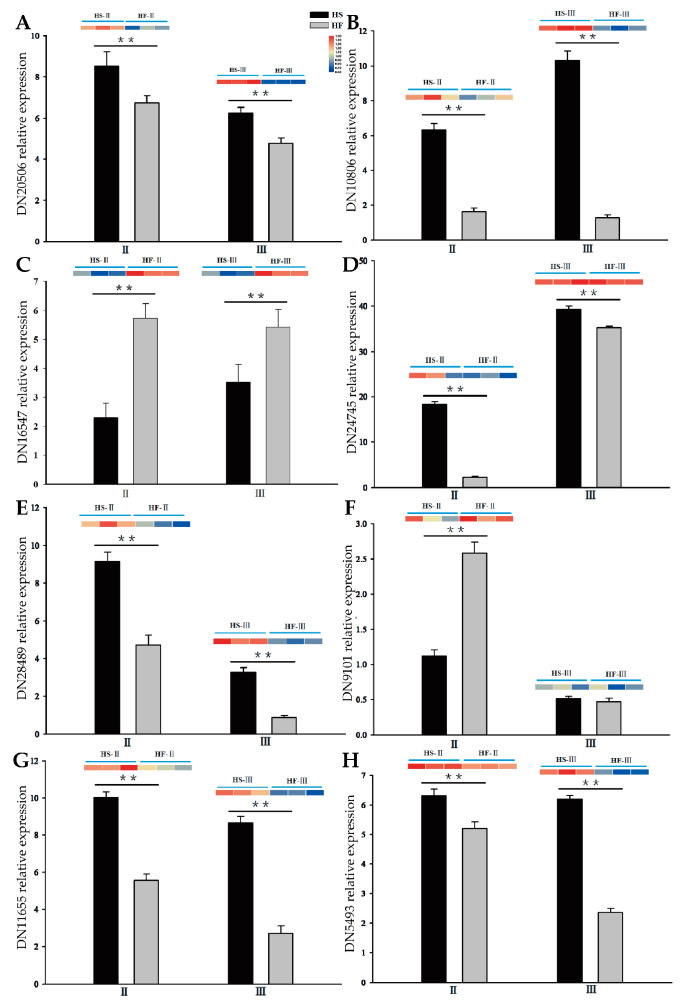
qRT–PCR results of hormone−related unigenes in the fertile line and sterile line during different anther development stages. (**A**): DN20506 (auxin response factor) expression level. (**B**): DN10806 (YUCCA) expression level. (**C**): DN16547 (cytokinin synthase) expression level. (**D**): DN24745 expression level. (**E**): DN28489 (gibberellin receptor) expression level. (**F**): DN9101 (gibberellin 2−beta−dioxygenase) expression level. (**G**): DN11655 (abscisic acid receptor) expression level. (**H**): DN5493 (abscisic−aldehyde oxidase) expression level. II, microspore mother cell stage; III, tetrad stage; HS, sterile hybrid; HF, fertile hybrid. Two independent experiments were performed, each with three technical replicates, and one experimental result is shown. Samples with different asterisks are significantly different: ** *p* < 0.01. The error bars indicate the mean ± SD (*n* = 3).

To verify the accuracy of the RNA−seq library, the above eight hormone unigenes were selected and examined using fluorescence quantitative real−time PCR (qRT–PCR). These unigenes were mainly involved in the synthesis and signal transduction of these four plant hormones. The qRT–PCR results of these eight selected unigenes were consistent with the expression trend from sequencing (Figure 9), indicating that these selected unigenes had a strong response to pollen abortion in the lily. The results of qRT–PCR verified the reliability of transcriptome sequencing.

**Figure 10:**
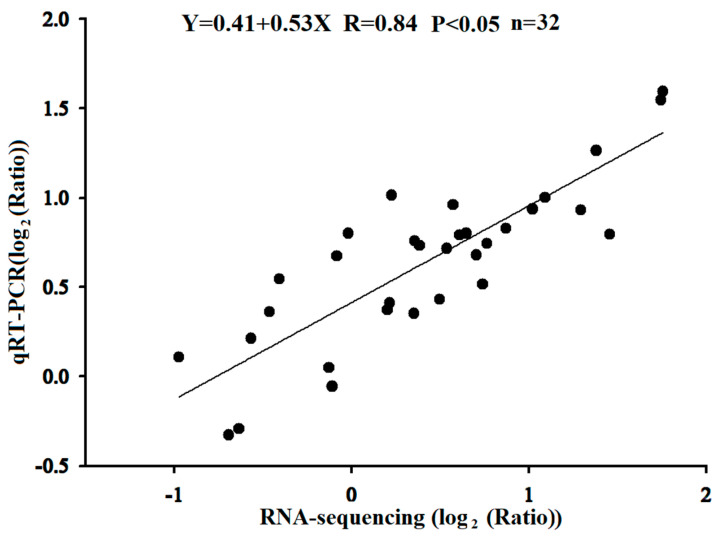
Correlation between qRT–PCR and RNA−sequence for the 8 selected genes. Each point represents a fold−change value of the expression level in HS compared with that in HF of the microspore mother cell stage and tetrad stage.

The correlation between the transcriptome sequencing and qRT–PCR results was comprehensively analysed, and the expression consistency of these eight unigenes was analysed by calculating the log2 value of the qRT–PCR result as the ordinate and the log value from the transcriptome sequencing as the abscissa. The results showed that the expression trends of the selected unigenes were essentially the same at the transcriptional sequencing level and the qRT–PCR level with a regression equation of Y = 0.41 + 0.54X (R = 0.84, *p* < 0.05) (Figure 10).

## 3. Discussion

Plant male sterility is the result of a combination of external environmental factors (light, temperature, air, gravity, etc.) and internal factors (gene expression regulation, hormones, cytokines, etc.), ultimately leading to metabolic disorders or abnormalities, such as disturbances in substances and energy, as well as deficiencies in nutrients and energy required for microspore development. These factors culminate in sterility [30].

Pollen−free lilies exhibit male infertility characterized by stamen degeneration with other plant flower parts developing normally; they cannot produce pollen due to malformed or absent stamens, leading to infertility. The tapetum cells of pollen−free lilies were different compared with those of the fertile hybrid in the microspore mother cell stage, which caused the microspore mother cells of the sterile hybrid to not be able to complete meiosis and enter the tetrad stage normally (Figure 1). The tapetum is located in the innermost layer of the anther chamber and mainly provides nutrients and related hormones during microspore development [31]. Early or delayed degradation of the tapetum can cause male infertility [32,33]. This result is similar to the function of *LoMYB80* which regulates the cessation of development and gradual degeneration of tapetum cells during the pollen mother cell stage in lilies [34], and is consistent with others reported in monocotyledonous plants [35,36]. Pollen abortion also begins mainly in the early stages of tetrad or microspore development in dicotyledonous plants [31,37]. In tomato (*Lycopersicon esculentum* Mill.), the mutant tapetum cells undergo mutations in the early stages of meiosis, further loosening and expanding the tapetum during the tetrad stage, leading to plasmolysis and the production of stamen degenerate male sterility [38]. In Chinese cabbage, pollen abortion mainly occurs during the tetrad stage and is generally due to abnormal expansion or vacuolization of anther cells, which leads to the loss of the nutrient supply needed for microspore development as well as compression of the tetrad, making it unable to dissociate uniformly, ultimately leading to male infertility [39]. Therefore, the development of pollen in the mother cell and tetrad stages should be regarded as a key stage for hormone−regulated pollen development, ultimately leading to the formation of pollenless lilies.

Regarding anther hormone content, male−sterile lily lines have shown metabolic disorders and hormonal imbalances compared to fertile lines. Proper nutrient accumulation, including starch, amino acids, and proteins, is crucial for pollen development [40]. Sugars play a significant role in anther development, and their content is closely related to pollen abortion. In cotton, for example, male−sterile lines displayed lower starch content than fertile lines [41]. Alterations in soluble proteins, which are crucial for microspore and anther development, can lead to metabolic disorders. Research has consistently demonstrated that there is a lower protein content in the leaves and flower organs of male−sterile lines compared to these parts in fertile lines during development [42]. This study found that the male−sterile lily line had lower contents of soluble sugars and proteins in the anthers, possibly due to blocked biosynthesis pathways. The proline content in the anthers of the male−sterile line remained low, while zeatin and ABA contents were correlated with proline content in fertile lines, suggesting that zeatin deficiency and increased ABA levels might impact proline content. Furthermore, the accumulation of MDA in plants reflects ROS toxicity and the degree of membrane lipid peroxidation, highlighting ROS accumulation in specific anther cells and tissues [43]. In this study, a positive correlation between MDA content and ABA in sterile line anthers was discovered, indicating that ABA might participate in MDA accumulation during development from the spore−forming cell stage to the tetrad stage. The increase in the content of this substance results from the abnormal anther development in the sterile line, possibly stemming from ROS accumulation due to the hindered anabolism of antioxidant substances in sterile line pollen [5]. Further investigation and verification are necessary to fully understand this phenomenon.

To discern alterations in endogenous plant hormone levels, as well as the types and quantities of differentially expressed genes (DEGs) during the degradation of lily anther tapetum cells, the DEGs related to hormone signal transduction pathways were identified in both fertile and infertile lily offspring. Employing advanced high−throughput sequencing analysis, we detected differentially expressed genes within genetically comparable backgrounds. Analysing the shifts in gene expression levels provides a foundational platform for gene function investigation and the analysis of male sterility in plants [6,44]. Further insights drawn from RNA sequencing underscored the 7400 and 10,719 DEGs in HS and HF during the microspore mother stage and tetrad stage, respectively. Notably, 3381 genes were shared between the two groups, with the DEGs primarily involved in regulating auxin, cytokinin, gibberellin, and abscisic acid metabolism.

The IAA content in lily anthers was consistently higher in the sterile line across all developmental stages. Enhanced expression of the ARF auxin signalling transduction−related gene *DN20506* in pollen correlated with male sterility. Auxin, a regulator during early stamen development [45], also impacts later stages, including anther dehiscence, pollen maturation, and chrysanthemum filament elongation in *A. thaliana* [46]. Remarkably, most enzyme−encoding genes (*DN10806*) pivotal to the transformation of TAA and YUCCA into IAA were consistently overexpressed in the sterile lily line (HS). These observations might be attributed to the elevated auxin content in the sterile line (HS), which sustains ARF overexpression, as many ARF genes are induced by auxin expression [47]. Differences in zeatin and GA4 levels between the sterile and fertile lines during the microspore mother stage were minimal. GA4, which is integral in various stages of plant growth and development, also governs tapetum cell development. The GA4 signalling pathway illuminates the multifaceted functions of GA4 in plant reproduction. The transcriptome data suggest that feedback control of GA4 signalling in male−sterile lilies modulates mRNA−level gene expression patterns during anther development. Notably, the ABA content in lily anthers during the tetrad stage was higher in the sterile line than in the fertile lines. Elevated auxin, increased ABA, and reduced gibberellin and cytokinin levels are often linked to male sterility in most plants [29]. Crucially, key genes associated with ABA synthesis, such as *NCED*, *SDR*, and *AAO*, were significantly upregulated in the sterile line. The expression of ABA receptor genes, including *PYR/PYL/RCARs*, as well as signal transduction genes *PP2Cs* and *SnRK2s*, increased notably during the tetrad stage, ultimately leading to tapetum layer degradation. Hormones are vital for pollen development, as hormone balance is pivotal for its success [48]. Typically, normally developing anthers possess very low amounts of ABA [49]. ABA triggered by biological or abiotic stress can impede pollen development [50]. Both IAA and ABA play pivotal roles in lily anther development. Studies on *Arabidopsis* and crops have similarly implicated auxin and ABA in stamen degradation [31,51].

**Figure 11:**
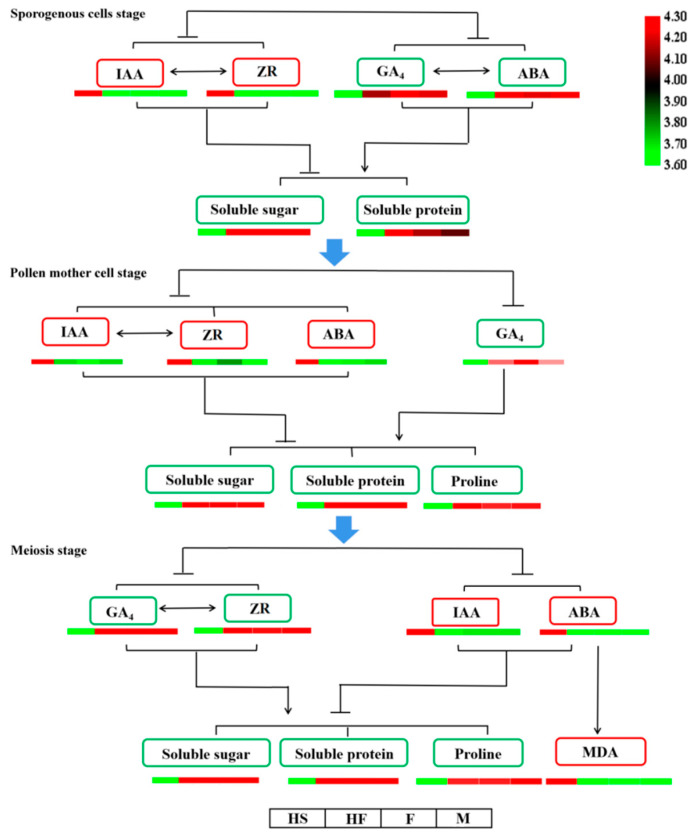
Model of the relationship between hormones and endogenous substances during the critical period of pollen−free formation in lily.

The interplay between hormones stands as the primary factor behind male sterility. According to the research results, a model diagram of the relationship between hormones and endogenous substances during the critical phase of pollen−free lily formation was devised (Figure 11). During the lily pollen spore−forming stage, the relative levels of IAA and ZR in the sterile line exceeded those in the fertile lines, while the relative levels of GA4 and ABA in the sterile line were lower, which correlated with reduced levels of soluble sugar and protein. A higher auxin content was also noted in the male sterile mutant *s12* of tomato and male−sterile lines of GMS and CMS rape seed [52]. In the microspore mother stage, the relative contents of IAA, ZR, and ABA in the sterile line were higher than those in the fertile lines, while the relative content of GA4 in the sterile line was lower than those in the fertile lines, which was related to the decreases in soluble protein, sugar and proline levels. Higher IAA and ABA contents were observed in CMS lines of capsicum [53]. At the tetrad stage, the relative contents of GA4 and ZR in the sterile line were lower than those in the fertile lines, which was related to the decreases in soluble protein, soluble sugar, and proline. Conversely, the relative contents of IAA and ABA in the sterile line were higher than those in the fertile lines, and the rise in ABA content led to MDA accumulation. Sugar beet endogenous IAA and GA levels differed from ABA levels at various developmental stages [54]. Wild−type *Arabidopsis* treated with exogenous GA and double mutants of *gai gra*, two repressors of GA signalling, exhibited loss of fertility [55]. Therefore, the metabolism of endogenous hormones in lily pollen is closely related to male fertility.

However, the physiological mechanism of male sterility in lily is complicated, and its occurrence is affected by changes in single plant hormones and the balance of various hormones with obvious characteristics of time and space. This study was limited to one or two periods of microspore abortion process, and did not consider the changes in endogenous hormones throughout the whole process of male infertility, which could cause the conclusion insufficient and systematic. Nonetheless, further research into how related genes govern hormones and endogenous substances to regulate male sterility in lily is our future direction. Moreover, the regulatory mechanism of fertility genes and downstream gene expression on hormones and endogenous substances in the process of male organ abortion in lily also becomes the focus of our research. The results of this study provide a foundational basis for explaining the regulation mechanism of hormones and endogenous substances in the development process of male sterility in lily, along with the exogenous hormone regulation of lily pollen fertility.

## 4. Materials and Methods

### 4.1. Test Materials

The experiment was conducted from April 2020 to April 2021 at the Lily Breeding Experimental Base (25°7′33″ N, 102°45′48″ E, altitude 1951.1 m), which is affiliated with the Institute of Flowers, Yunnan Academy of Agricultural Sciences. Following several years of deliberate breeding efforts, a distinct hybrid population of Oriental lily emerged. This hybrid population’s parent plants were fertile, and the hybrid offspring showed a typical Mendelian genetic separation ratio.

In the greenhouse, 30 of the parents (maternal: F, and paternal: M) were planted, and 30 of the fertile offspring (HF) and sterile offspring (HS) were selected. The planting density was 12 cm in row spacing and 15 cm in plant spacing. The anthers of the sterile and fertile lines in the sporogenous cell stage, microspore mother cell stage and tetrad stage were collected, quickly frozen in liquid nitrogen after sampling, and stored at −80 °C for later use.

### 4.2. Observation of Lily Anther Morphology

After bud emergence, plants with the same growth and developmental level were selected, and the anthers at the sporogenous cell stage, microspore mother cell stage, tetrad stage, and microspore stage were stripped according to the standard of correlation between lily organ morphology and anther development. Lily anthers were fixed in FAA fixing solution (100 mL containing 5 mL of 38% methanol, 5 mL of acetic acid, and 90 mL of 70% alcohol), and the slices were made by the conventional paraffin section method with a thickness of 3 μm. Then, the slices were stained with 1% safranin−O for 1 h, washed with distilled water, discoloured and then counter−stained with 0.5% fast green for 1 min (G1031; Servicebio, Wuhan, China). Finally, the sections were observed and photographed under an optical microscope (YS100; Nikon, Tokyo, Japan).

### 4.3. Examination of the Endogenous Hormone Content

The contents of the growth hormones indole−3−acetic acid (IAA), abscisic acid (ABA), zeatin riboside (ZR), and gibberellic acid 4 (GA4) were measured in anther samples from both sterile and fertile lily strains. The experiment was repeated 3 times for each group, and the mean value was taken for statistical analysis. Amongst them, the plant hormones IAA, ABA, and GA4 were determined by high−performance liquid chromatography (nano−LC−ESI−Q−TOF−MS) [56]. Cytokinin and zeatin were determined using tandem mass spectrometry (PMME/HILIC/ESI−MS/MS) [57].

### 4.4. Determination of the Contents of Endogenous Substances

The soluble sugar, soluble protein, proline and malondialdehyde (MDA) contents were determined. Soluble sugar was determined by the anthracene colorimetric method, soluble protein by the Coomassie colorimetric method and proline (Pro) by the hydrindene solution colorimetric method [58]. The MDA content was determined by the thiobarbiturate method [59].

### 4.5. Transcriptome Sequencing of Aborted Pollen

The anthers of HS and HF microspores at the mother cell stage (II) and tetrad stage (III) were selected and quickly frozen in liquid nitrogen, with three replicates (II−HS1, II−HS2, and II−HS3; III−HS1, III−HS2, and III−HS3; II−HF1, II−HF2, and II−HF3; III−HF1, III−HF2, and III−HF3). Total RNA was extracted by an RNA extraction kit (RN40; Aid lab Biotechnologies, Beijing, China). The library was sequenced by the BGISEQ−500 platform (China Ouyi Biotechnologies Co., Ltd., Shanghai, China). After the readings were filtered, the remaining clean reads were assembled from scratch using Trinity. The TGI Clustering Tool (TGICL) was used to cluster transcripts to remove discrepancies and obtain unigenes. These data were then combined with the nonredundant Protein Sequence Database (NR), the SwissProt database, the Kyoto Encyclopedia of Genes and Genomes (KEGG) database, the Gene Ontology (GO) database, the Clusters of Orthologous Groups for eukaryotic complete genomes (KOG), Evolutionary Genealogy of Genes: Non−supervised Orthologous Groups (eggNOG), and the Protein family database (Pfam) to annotate the transcripts. The expression level of each single gene was normalized to fragment (FPKM) values of transcripts read per kilobase per million mappings. The differentially expressed genes were then filtered and identified using calibrated criteria with *p* values < 0.01 and log2 (multiple variation) > 1 or < −1.

### 4.6. qRT–PCR for Hormone−Related Genes

Real−time quantitative PCR (qRT–PCR) was used to verify the reliability of transcriptome sequencing, and specific primers for selected genes were designed using Primer Express software version 3.0. The signal was monitored by the CFX Connect real−time system (Bio−Rad, Tokyo, Japan) using the 2^−ΔΔCt^ method. The amount of lily ACTIN in each sample was used to normalize the amount of each target mRNA, with three biological replicates set for each template [6]. The primers used are listed in Supplementary Table S1.

### 4.7. Data Processing

Microsoft Excel was used to sort the data and calculate the mean values and variance. SPSS 20 statistical software (SPSS Inc., Chicago, IL, USA) was used for single factor difference analysis, and Duncan’s test was used to analyse the differences amongst the treatments at the level of *p* < 0.05. SigmaPlot 10.0 was used for data plotting.

## Figures and Tables

**Table 1:** Screening genes and corresponding KEGG description.

	Gene	KEGG Description
IAA	DN20506_c0_g1_i2_4	Auxin response factor
	DN10806_c0_g1_i1_3	Indole−3−pyruvate monooxygenase
Cytokinin	DN24745_c1_g2_i2_2	Pseudohistidine−containing phosphotransfer protein
	DN16547_c0_g1_i1_2	Cytokinin synthase
GA	DN9101_c0_g1_i2_5	Gibberellin 2−beta−dioxygenase
	DN28489_c0_g1_i1_1	Gibberellin receptor
ABA	DN11655_c0_g1_i1_2	Abscisic acid receptor
	DN5493_c0_g1_i2_3	Abscisic−aldehyde oxidase

## Data Availability

Data are contained within the article and Supplementary Materials.

## Supplementary Materials

The following supporting information can be downloaded at: https://www.mdpi.com/article/10.3390/plants12223804/s1, Table S1: Primers used in the qRT−PCR.
