# Supplementary material for: Disruption of the Contents of Endogenous Hormones Cause Pollen Development Obstruction and Abortion in Male-Sterile Hybrid Lily Populations
Source: Plants (Basel). 2023 Nov 8;12(22):3804. doi: 10.3390/plants12223804 (PMC10674860; doi:10.3390/plants12223804)
Supplement: Supplementary file 1 [file plants-12-03804-s001.zip › plants-2612862-supplementary.pdf]

Table S1. Primers used in the qRT-PCR.

Title 1	Title 2	Direction	Primer sequence (5'–3')
IAA	DN20506_c0_g1_i2_4	Forward	ATGGCAACAATTGAGGAGCA
		Reverse	TTGTTAGCTGCAAACCCTCC
	DN10806_c0_g1_i1_3	Forward	TCCGATCCTGGAAATCAAGC
		Reverse	TGGGAAACCGTTTCTCTTGT
Cytokinin	DN24745_c1_g2_i2_	Forward	ACTCTAACTTGCAACGCCAG
		Reverse	TAGCACGAGTAGCTCTTTCG
	DN16547_c0_g1_i1_2	Forward	GAGAATTTTACTCCGCACCG
		Reverse	CTCTTCCAAGAAACGCCCAA
GA	DN9101_c0_g1_i2_5	Forward	TACGAAAACAGCATGAAGGG
		Reverse	AGGCCCACTTTATATCCTGT
	DN28489_c0_g1_i1_1	Forward	GCTGTGGTGATTTCGGTCAA
		Reverse	GGTCGTCACAAAGTCCTTGA
ABA	DN11655_c0_g1_i1_2	Forward	AAGCCCTCCGTTCAGCAC
		Reverse	GCGCCAACGACTGGAGAT
	DN5493_c0_g1_i2_3	Forward	GAGCAGTTGGAGGAGAAGCT
		Reverse	ACCTTTCGACATTGTCCAGC
Actin	Actin-F	Forward	GCCTCCGATCAATCGAAAGT
	Actin-R	Reverse	GAATGCCAGCAGCTTCCATC
